# Electrically driven nanogap antennas and quantum tunneling regime

**DOI:** 10.1515/nanoph-2023-0099

**Published:** 2023-06-20

**Authors:** Claire Deeb, Johann Toudert, Jean-Luc Pelouard

**Affiliations:** Almae Technologies, Route de Nozay, 91460 Marcoussis, France; Ensemble3 Centre of Excellence, Warsaw, Poland; Centre for Nanoscience and Nanotechnology, CNRS, Université Paris-Saclay, 10 Bvd T. Gobert, 91120 Palaiseau, France

**Keywords:** hot carriers, inelastic electron tunneling, nanogap antennas, photon emission, quantum regime, tunnel junction

## Abstract

The optical and electrical characteristics of electrically-driven nanogap antennas are extremely sensitive to the nanogap region where the fields are tightly confined and electrons and photons can interplay. Upon injecting electrons in the nanogap, a conductance channel opens between the metal surfaces modifying the plasmon charge distribution and therefore inducing an electrical tuning of the gap plasmon resonance. Electron tunneling across the nanogap can be harnessed to induce broadband photon emission with boosted quantum efficiency. Under certain conditions, the energy of the emitted photons exceeds the energy of electrons, and this overbias light emission is due to spontaneous emission of the hot electron distribution in the electrode. We conclude with the potential of electrically controlled nanogap antennas for faster on-chip communication.

## 1 Introduction

Since the nineties, the field of plasmonics has grown by merging concepts from the fields of material science and RF antennas. This has led to pioneering results where suitably designed metal nanostructures behave like resonant antennas at optical frequencies, thus enabling efficient coupling of visible or near-infrared light to matter in deeply subwavelength volumes [1]. Remarkably, surface plasmons in metal nanostructures enable strongly enhanced electric fields in their nanoscale surrounding, which have proven useful, for example, to locally enhance Raman and fluorescence signals or for photothermal conversion [2].

Since then, achieving plasmon resonances and maximum enhanced fields at selected wavelengths has been the focus of many studies, which have been greatly supported by modern nanofabrication and nanocharacterization techniques to study the optical properties of metal nanostructures, either isolated or grouped as dimers, trimers, oligomers, or supracrystal films [3–5]. In particular, it was shown that classical electromagnetic interaction between closely packed metal nanostructures, especially dimers, may lead to a more intense local electric field within the gap, compared with an isolated nanostructure.

This result has motivated researchers to explore the interaction between metal nanostructures separated by a very small, nanometric gap. This exploration has revealed the onset of quantum physics effects that strongly affect the plasmonic and electric field enhancement of the interacting nanostructures. When the distance between nanostructures is sufficiently small, electrons may tunnel between them. Upon applying a bias voltage, this endows them with electroluminescence properties that are appealing for designing electrically driven, nanoscale and ultrafast light emission or detection devices [6, 7].

To produce efficient and practical devices, a clear overview of the fundamental optoelectronic properties, and production possibilities and constraints of such nanostructures, also called “nanogap antennas”, is needed. With such aim in mind, this article reviews recent works on nanogap antennas.

In Section 2, the plasmonic and local electric field enhancement properties of nanogap antennas are discussed from a theoretical point of view. For a clear understanding, the case of a metal dimer with a gap distance broadly tuned to cover both the classical and quantum regimes is considered.

In Section 3, experimental works showing the measured plasmonic, local electric field, Raman and fluorescence enhancement properties of nanogap antennas in the classical and quantum tunneling regimes are reviewed, and their findings are compared with theoretical expectations.

In Section 4, experimental findings related to the electroluminescence of nanogap antennas, occurring thanks to inelastic electron tunneling, are presented. This section provides an overview over a broad range of reported nanogap antennas, differing by their structure (metal-insulator-metal structures, nanostructure dimers, vertically organized structures) or their chemical nature (“alternative” materials beyond metals). Performance indicators, such as the electroluminescence quantum efficiency, spectrum, and electrical tunability, are systematically reported.

Section 5 makes an emphasis on electroluminescent nanogap antennas harnessing a different light-emission mechanism, which involves hot carrier processes. Such type of antennas promises a broader spectral tuning and higher quantum efficiency than those involving inelastic electron tunneling.

Section 6 briefly discusses recent attempts to produce practical, miniaturized, and tunable light-emission devices based on nanogap antennas.

## 2 Theory of nanogap antennas: from classical to quantum regime

Concentrating light into nanoscale dimensions was made possible through structuring sub-wavelength metal objects that can support collective oscillations of electrons when interacting with electromagnetic radiation. This localization effect produces great field enhancements of several orders of magnitude compared to the incident value. Such plasmonic enhancements significantly boost the efficiencies of light–matter interactions at the nanoscale and have impacted applications such as energy harvesting [8], biomedicine [9, 10], photo-detection [6, 11, 12] and plasmon nanolasers [13–17]. Compared to single nanoparticles (NPs), dimer structures – two nanoparticles separated by a nanoscale gap – were proven particularly effective in squeezing light into deep-wavelength volumes [17–19]. Gaps formed between metal surfaces control the coupling of localized plasmons, thus allowing gap-tuning targeted to exploit the enhanced optical fields for different applications [20–23].

Classical electrodynamic approaches are frequently used to describe the plasmonic response of metal gap structures with gaps on the order of few nanometers [24, 25]. Using these classical approaches, the different materials are separated by sharp boundaries. The optical properties of materials are described by a frequency- and position-dependent linear dielectric function *ɛ*(*w*, *r*) [26]. This theory predicts a monotonic increase in the electric field enhancements with decreasing gap distance, prompting the production of plasmonic structures with sub-nanometer gaps.

**Figure 1:**
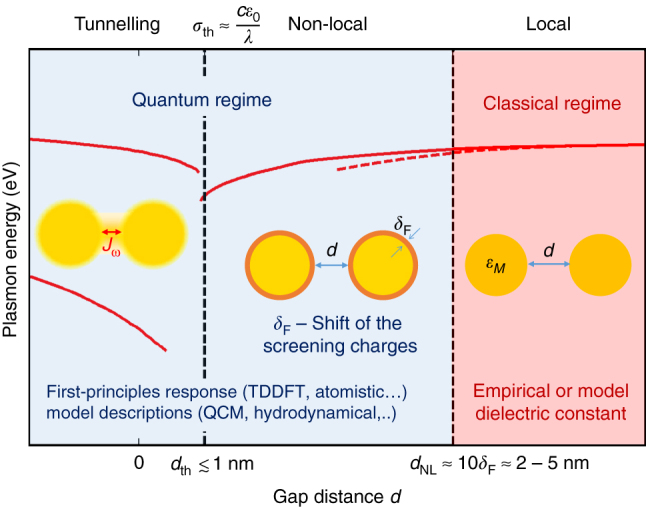
Influence of quantum mechanical effects on plasmon resonances of nanogap structures. Three regimes are predicted by classical (red dashed line) and quantum calculations (solid line) and illustrate the energies of the plasmonic modes of a dimer of spheres as a function of the gap distance *d*. A classical local regime (*d* ≥ *d*_*NL*_ ≈ 2–5 nm) where local Maxwell equations correctly describe the red-shift of the gap plasmon modes. A non-local quantum regime (*d*_th_ < *d* < *d*_*NL*_) where the screening charges with respect to the geometrical boundaries of the metal leads to an effective modification of the metal interface boundaries, producing an effective gap distance that differs from the geometrical value. A tunneling regime, separated from the non-local quantum regime by a threshold tunnel distance *d*_th_, where the conductivity of the junction is large enough to strongly perturb the optical response of the structure. Reproduced from ref. [27] with permission; copyright 2016 Nature Publishing.

However, as the gap distance enters the nanometer and then the sub-nanometer scale, classical electrodynamics fail to describe the optical response of nanogap structures, where quantum effects become important owing to non-local screening (also called spill-out effect in the literature) and electron tunneling across the gap [28–33]. The quantum nature of electrons and the non-local screening strongly alter the optical response and lead to deviations from the classical description as shown in Figure 1. In this quantum regime, surface charges are corroborated with a frequency-dependent distance parameter *δ*_*F*_, the Feibelman parameter, which defines the actual position of the screening charges with respect to the geometrical boundaries of the metal, leading to an effective gap distance that differs from the geometrical value [27]. Moreover, the spatial profile of the screening charges is described by a smooth transition of the electronic densities at the interface. Electron tunneling between the metal surfaces is another key quantum feature that the classical local model fails to capture.

Tunneling current across the nanogap induces a conductive contact prior to the direct geometrical contact, which reduces the Coulomb coupling between charges of opposite signs on the two sides of the nanogap and strongly affects the optical response of the nanogap structures [34]. The impact of these quantum mechanical effects on the optical response of nanogap structures is schematically illustrated in Figure 1. This figure shows the different regimes for the plasmon resonances of a pair of particles as a function of the gap distance *d*. For large gap distances (≥2–5 nm), the structure is in the classical regime, and its optical response can be correctly described using Maxwell equations with gap plasmon modes red-shifting (due to higher effective index of the mode) as the gap is decreased.

As the gap becomes smaller than a few nanometers with a sufficiently small conductance between the particles, the optical response becomes dominated by the non-local screening quantum effect. The non-local screening leads to deviations of plasmon resonances from the classical (dashed line) to the quantum regime (solid line). As the gap distance continues to decrease and becomes smaller than the threshold tunneling-distance *d*_th_, the tunneling-induced conductance becomes large enough to allow a fraction of surface charges to tunnel across the gap. The plasmon resonance of the structure is strongly affected by the *ac* current tunneling across the junction, resulting in a progressive extinction of the red-shifting gap plasmon mode and a gradual emergence of the blue-shifting charge-transfer plasmon (CTP) mode.

In this context, various semi-classical models have been developed to partially account for non-classical features [35]. For example, some of these descriptions illustrate the intrinsic nonlocality of the metals by combining classical electrodynamics and hydrodynamic descriptions of the induced charges [28, 30, 36–38]. Moreover, other semi-classical approaches account for the spill-out of the induced electron density and Landau damping by incorporating ab initio quantum surface-response corrections at the metal–dielectric boundaries. This can be achieved using Feibelman parameters obtained from quantum calculations [26, 39–42]. These models have predicted that non-classical phenomena can substantially influence the optical interaction in a nanogap structure. Recently, fully quantum calculations based on the time-dependent density functional theory (TDDFT) have been proposed to capture all the quantum effects affecting the interaction in nanogap antennas and to establish the validity of the different semi-classical models [43].

## 3 Experimental demonstration of quantum mechanical effects in nanogap antennas

Interest in developing theoretical models for addressing quantum mechanical effects in nanogap structures [23, 26, 29, 40, 44–48] is triggered by the tremendous progresses in nanofabrication and experimental techniques to produce and measure such structures. Previous efforts for fabricating nanogap antennas typically relied on top–down approaches such as electron beam lithography (EBL) or bottom–up approaches based on self-assembled nanoparticles. The resolution of a single exposure EBL is limited by electron scattering in the substrate, allowing gap distances down to ∼2 nm only. In bottom–up techniques, the gap distances achieved are limited by the size of the surfactant molecules used to fabricate the plasmonic assemblies. In this section, we summarize some recent key approaches that employed plasmonic structures with sub-nanometer gaps and allowed the experimental observation of tunneling and non-local effects.

### 3.1 Non-local effects in plasmonic air-nanogap antennas

**Figure 2:**
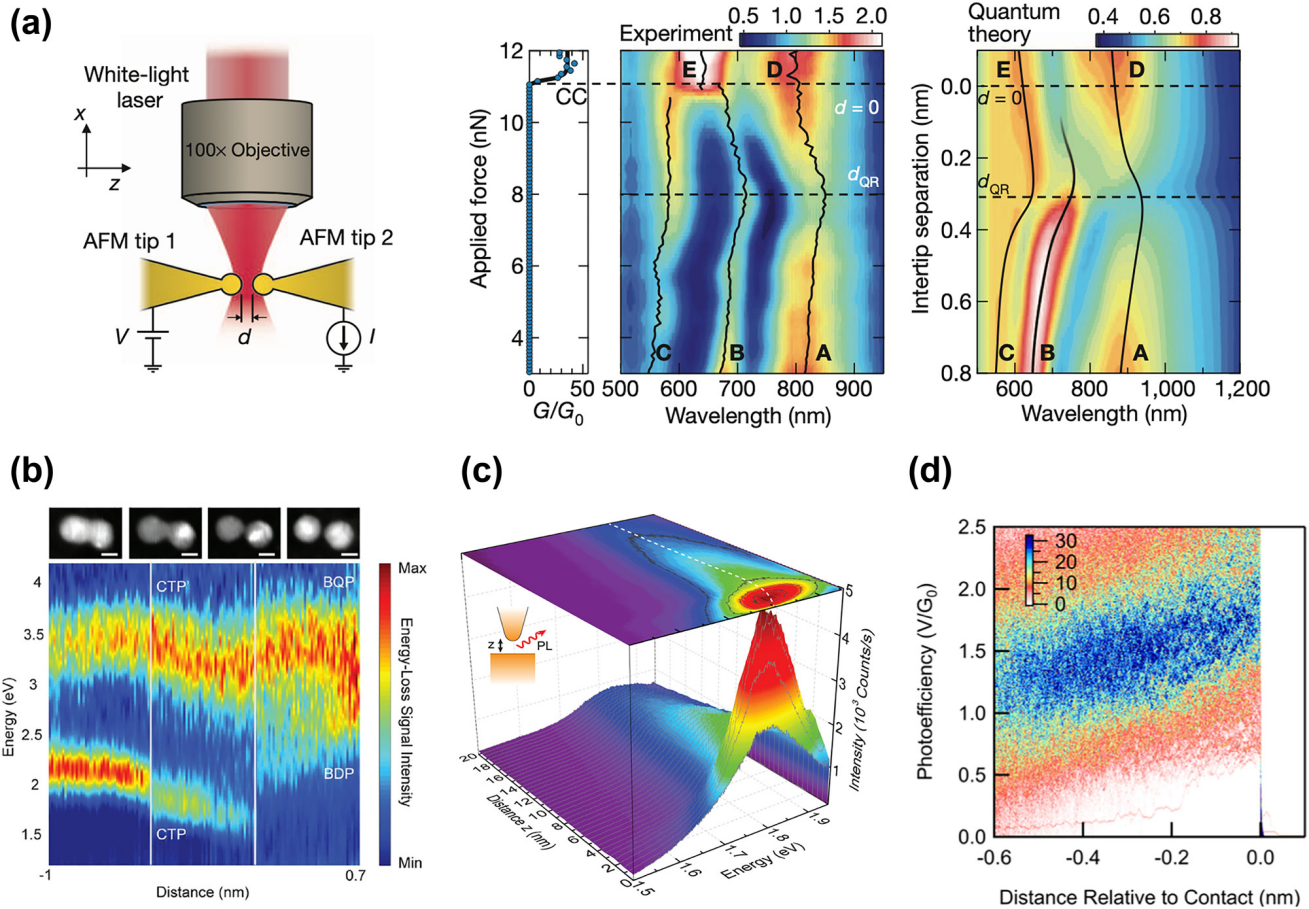
Experimental demonstration of quantum tunneling in sub-nanometer plasmonic cavities. (a) Schematic of plasmonic cavity for simultaneous optical and electrical measurements in a dual AFM tip configuration. Simultaneously measured electrical conductance *G*/*G*_0_ and dark-field scattering spectra of the plasmonic cavity with increasing force applied to the tip cavity. Calculated total scattering intensity from a plasmonic cavity system incorporating quantum mechanical tunneling. Reproduced from ref. [49] with permission; copyright 2012 Nature Publishing. (b) Electron energy-loss spectroscopy (EELS) signals of a 9-nm diameter silver dimer collected during particle motion triggered by a STEM-EELS probe. STEM images collected at the beginning and end of each scan (indicated by solid vertical lines) illustrate the separation distances (+0.7, <0.27, −0.3, and −1.0 nm). Image scale bar, 5 nm. Reproduced from ref. [50] with permission; copyright 2012 American Chemical Society. (c) Evolution of Au tip PL spectra in scanning tunneling microscope-based configuration as a function of distance to the Au-surface, *z*. Pronounced field enhancement is followed by rapid quenching for *z* < 2 nm. Reprinted with permission from ref. [51]; copyright 2014 American Chemical Society. (d) Two-dimensional histogram of photoefficiency versus distance relative to contact formation for light emission of wavelengths 850 nm and longer. Reprinted with permission from ref. [52]; copyright 2022 American Chemical Society.

Savage et al. [49] achieved gap distances in the tunneling regime by bringing two gold-nanoparticle-terminated atomic force microscope (AFM) tips to close proximity using a piezoelectric stage. Gap distances in the sub-nanometer regime were produced, with a *dc* tunneling conductance *G* across the gap exceeding the quantum conductance *G*_0_ = 2*e* ^2^/*h* (where *e* is the elementary charge and *h* is the Planck’s constant) for tips in conductive contact. The dark-field scattering spectra recorded as a function of the reducing gap distance are shown in Figure 2(a). For *d* > *d*_*QR*_, with *d*_*QR*_ the threshold distance at which charges induced by quantum tunneling overcome the near-field capacitive interaction between plasmons, spectra are dominated by the near-field interaction of the cavity localized charges and plasmons couple according to classical models. As the structure separation decreases below a critical size *d* ∼ *d*_*QR*_, plasmons interactions enter the quantum regime characterized by a gradual evolution of the bonding modes into CTP modes. Quantum tunneling charge transfer was found to screen the localized surface plasmon charges, therefore decreasing the enhanced fields and reducing the plasmonic coupling. These results agree well with the predictions of the quantum corrected model (QCM, quantum approach incorporating quantum mechanical effects for nanogap plasmons within a classical electrodynamic framework) of Esteban et al. [26, 40] and recent experiments by Scholl and colleagues [50].

Scholl et al. observed the plasmon resonances of coupled metal nanoparticles as their gap size was reduced down to sub-nanometer dimensions (Figure 2(b)). As separations were reduced from 7 nm, the dipolar peak exhibited a redshift consistent with classical calculations. However, for gaps smaller than ∼0.5 nm, this mode showed a reduced intensity, implying that field enhancements will saturate around this gap separation, which is again consistent with quantum theories that incorporate electron tunneling. As the particles overlapped, the dipolar mode disappeared and was substituted by a dipolar charge transfer mode [50].

Additionally, Kravtsov et al. explored the photoluminescence (PL) of a coupled plasmonic structure consisting of a sharp AFM gold (Au) tip placed on the top of an ultra-flat templated-stripped Au film [51]. Field-enhanced behavior accompanied with a maximum PL intensity dominated until the onset of quantum coupling (gap distance ∼1.5 nm) dramatically reduced field enhancement and emission intensity (Figure 2(c), inset: scheme illustrating the experimental configuration). The PL spectral peaks blue-shifted for smaller gaps, an additional indication of the emergence of electron tunneling.

Recently, the electroluminescence and conductance of Au tunnel junctions have been simultaneously probed [52]. As the gap size was reduced, the plasmonic enhancement increased for junctions biased between 1.4 and 1.8 V, which is consistent with the behavior of charge transfer plasmons. At biases above 1.9 V, the plasmonic enhancement decreased with the decreasing gap, showing quenching due to tunneling as shown in Figure 2(d), which is in remarkable agreement with the trends observed for high energy plasmons in scattering experiments.

### 3.2 Non-local effects in molecule-loaded-nanogap antennas

Ciraci et al. studied the plasmon resonances of the particle-on-film structure, with the gap being formed using a molecular dielectric layer. The peak wavelength of the plasmonic resonance was found to deviate from the predictions of the local classical model for gap distances less than ∼3 nm [53]. These deviations can be accounted for by the hydrodynamic model that incorporates non-locality effects.

Zhu et al. also measured the surface enhanced Raman scattering (SERS) enhancement of thiophenol molecules adsorbed on a pair of nanoparticles as a function of the gap distance. The emergence of electron tunneling at optical frequencies for gaps below a threshold tunnel-distance limits the maximum achievable plasmonic enhancement and induces a quenching of the SERS enhancement [54]. QCM calculations were found to be consistent with the experimental data, supporting that quenching of near-field enhancement was due to electron tunneling.

**Figure 3:**
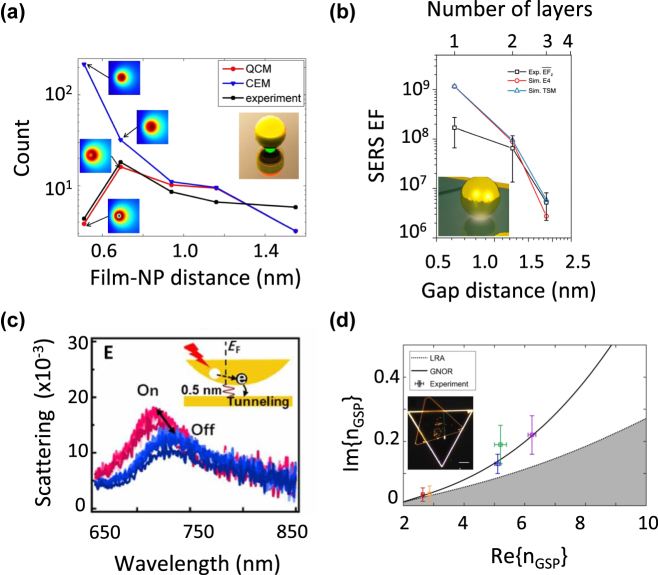
Experimental observation of electron tunneling effects in molecule-loaded-nanogap antennas. (a) Comparison between quantum corrected model, classical electromagnetic model, and experiment for a particle-on-mirror configuration. Insets show electric field intensity distribution at Au film surface just below the NP for *λ* = 1570 nm. Reprinted from ref. [55] with permission; copyright 2014 American Chemical Society. (b) Measured and simulated field enhancement as a function of the gap distance in a nanoparticle-on-film structure. The simulated SERS EFs based on classical theories match well with the measured SERS EFs for gap distances > 1.24 nm, while significantly overestimate the field enhancement at shorter gap distance. Reproduced from ref. [56] with permission; copyright 2018 Nature Publishing. (c) Change of plasmon scattering peak with CW laser (641 nm, 10 μW) on and off for many cycles. Inset scheme illustrates the light-induced hot electron tunneling mechanism, which changes the conductance of the nanogap. Reproduced from ref. [57] with permission; copyright 2022 American Association for the Advancement of Science. (d) Parametric plot of the effective-mode index *n*_GSP_ (at the excitation wavelength *λ* = 1550 nm) for varying dielectric gap thickness: calculated using local-response approximation (LRA, dashed curve), generalized non-local optical response model (GNOR, solid curve) and experimentally obtained data. Colors of the indicated points on the curves and experimental data points correspond to 2, 3, 5, 10, and 20 nm gap thicknesses. Inset: Dark-field optical image of the full structure, scale bar 10 μm. Reproduced from ref. [58] with permission; copyright 2022 Nature Publishing.

Moreover, Hajisalem et al. probed the onset of the quantum tunneling regime by observing the local field intensity in sub-nanometer self-assembled monolayer gaps of particle-on-film structures [55]. A sudden drop in the third harmonic generation occurred for double the gap size of past studies because of the reduced barrier height of the self-assembled monolayers (Figure 3(a)).

Chen et al. developed a MoS_2_ spaced nanoparticle-on-mirror plasmonic antenna, with layered MoS_2_ as a two-dimensional atomic crystal serving as the gap for probing field enhancement within the gap [56]. Measured SERS enhancement factor (EF) showed great agreement with calculations from pure classical electromagnetic theory for gap distances > 1.24 nm. For a gap of 0.62 nm, the emergence of quantum mechanical effects yielded a maximum field enhancement of 114-fold, 38.4 % lower than classical predictions, demonstrating that classical model significantly overestimates the field enhancement for sub-nanometer gap distances as shown in Figure 3(b).

Recently, supramolecular systems made of oligoamide sequences were used to reversibly switch the gap plasmons of Au nanoparticles on mirror between classical and quantum tunneling regimes via supramolecular interactions [57]. Plasmonic hot electron tunneling driven by laser excitation was proved to blue shift the dipolar mode of gap plasmons because of the increased conductance in the nanogaps. The plasmon peak switched back to its original value when the laser was turned off as illustrated in Figure 3(c). These plasmon shifts fitted perfectly with both classical and quantum-corrected models, suggesting that the quantum limit is approximately around 0.6 nm.

Also, non-local effects have been investigated in propagating gap surface plasmon modes in ultrathin metal–dielectric–metal planar waveguides, exploiting monocrystalline gold flakes separated by atomic-layer-deposited aluminum oxide [58]. Scanning near-field optical microscopy (SNOM) was used to directly access the near-field of such confined gap plasmon modes. Scattering-type-SNOM measurements from samples with different gap thicknesses revealed signatures of gap-dependent non-classical broadening, which was well-described by the generalized hydrodynamic model of plasmonics (Figure 3(d)). Thus, quantum non-local corrections should be taken into account when treating extremely confined gap plasmon modes.

## 4 Inelastic electron tunneling

In the previous sections, nanogap antennas with gap distances changing from the nanometer scale to the sub-nanometer scale were introduced and the difference between classical and quantum tunneling regimes was explained. The case of a metal dimer with a gap distance broadly tuned to cover both the classical and quantum regimes was considered. The plasmonic electric field enhancement properties of nanogap antennas were discussed from a theoretical point of view. Then, some recent key approaches that employed plasmonic structures with sub-nanometer gaps (air- and molecule-loaded) and allowed the experimental observation of tunneling and non-local effects were reviewed. These experimental findings were then compared to theoretical expectations.

In this section, experimental findings related to the electroluminescence of nanogap antennas, occurring based on inelastic electron tunneling, will be presented. Other physical mechanisms including hot-electron injection involved in light emission process will be briefly discussed. Then, an overview over a broad range of reported nanogap emitting-light-antennas, differing by their structure (metal–insulator–metal structures, nanostructure dimers, vertically organized structures) or their chemical nature (“alternative” materials beyond metals) will be provided. Performance indicators, such as the electroluminescence quantum efficiency, spectrum, and electrical tunability, will be systematically reported.

### 4.1 Electroluminescence mechanism

#### 4.1.1 Inelastic electron tunneling, plasmon excitation and outcoupling

**Figure 4:**
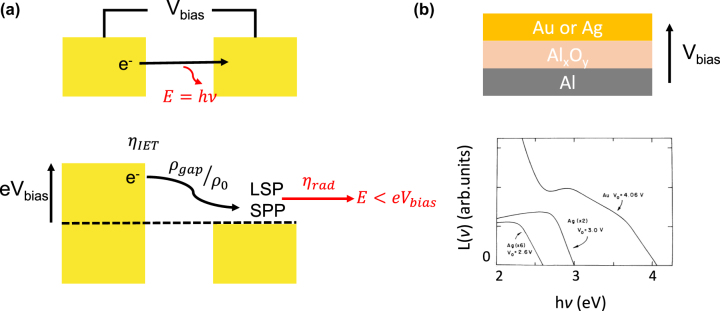
Electroluminescence induced by inelastic electron tunneling in a nanogap structure. (a) Schematic representation of a metal-insulator (void)-metal nanogap structure and of the light emission process. Upon applying a voltage *V*_bias_, electron inelastic tunneling occurs and leads to the emission of a photon. This emission process can be decomposed into 3 steps: (i) tunneling (efficiency *η*_IET_), (ii) coupling with plasmons (Purcell effect, efficiency *ρ*_*p*_/*ρ*_0_), and (iii) plasmon outcoupling (efficiency *η*_rad_). The energy of emitted photons depends on *V*_bias_ and is smaller than *V*_bias_ at 0 K. (b) Tuning of the electroluminescence spectrum with *V*_bias_ in Au (or silver)/aluminum oxide/aluminum junction. The cutoff at e*V*_bias_ is clearly seen. Reproduced from ref. [62] with permission; copyright 1976 American Physical Society.

When a bias *V*_bias_ is applied between two metal electrodes separated by an insulating nanogap, the Fermi level of one of the electrodes is raised by an energy e*V*_bias_ with respect to the other, as shown in Figure 4(a). If the nanogap is thin enough (at most, a few nm), some electrons tunnel through it, a fraction of them elastically and the other fraction inelastically. In the inelastic scheme, energy may be conserved through photons emission from the nanogap structure. The most frequently reported mechanism for such light emission, which is depicted in Figure 4(a), is the following: (i) each of the inelastically tunneling electrons may generate one photon that radiates to the far-field or one surface plasmon at the metal surface. Depending on the morphology of the electrodes, the thereby generated plasmons may propagate at the interface (surface plasmon polariton, SPP) or be localized (localized surface plasmon, LSP). (ii) Plasmons may outcouple into photons released to the far-field. The probability of such outcoupling depends on the electrode morphology.

The mechanism involving inelastic electron tunneling and plasmons in a single-electron process has been claimed to be the dominant path for electrically driven light emission when the nanogap conductance *G* is smaller than the quantum of conductance *G*_0_ (*G* « *G*_0_). However, it has also been observed in some studies where *G* was close from *G*_0_. This mechanism endows the nanogap structure with electroluminescent properties that are appealing for devices harnessing the properties of plasmons such as nanoscale light sources and sensors. In this context, it is particularly relevant that the process of inelastic electron tunneling occurs at the femtosecond timescale, i.e. much faster than radiative recombination in semiconductors. This would be promising to achieve ultrafast modulation of the electroluminescence signal.

Other mechanisms for light emission rather than relying on an inherently inelastic tunneling effect in the gap have also been demonstrated. Uskov et al. proposed a light source utilizing light emission by electron passing ballistically through a nanoconstriction [59]. When a voltage is applied across the nanoscale constriction used as the active element of the optical antenna, electrons flow through the contact quasi-ballistically, gain energy and transfer it to excited plasmon modes, provided that both energy and momentum conservation is maintained.

Light emission based on coherent surface plasmon amplification due to the tunneling gain was also discussed [60]. A detailed comparison of the surface plasmon gain due to the inelastic tunneling and loss due to the interband and Drude absorption shows the possibility of the net gain in sufficiently thin tunnel structures. Mechanisms describing photon emission out of nanogap antennas at energies exceeding the applied bias will be discussed in Section 5.

#### 4.1.2 Electroluminescence spectrum and external quantum efficiency

In the context of such applications, key performance indicators are the electroluminescence spectrum of the nanogap structures and the corresponding external quantum yield (*η*_ext_ = number of emitted photons/number of tunneling electrons). To give qualitative insights into such indicators, one can relate them to the underlying microscopic mechanisms. The electroluminescence is often modeled as three-step process [61], depicted in Figure 4(a):

(i) Inelastic electron tunneling by quantum current fluctuations. This generates radiative dipoles in the nanogap. With E being the emission energy, the efficiency *η*_IET_(*E*) of this process depends, at 0 K, on the fraction of electrons that tunnel inelastically and on (e*V*_bias_ – *E*). If *E* > e*V*_bias_, *η*_IET_ = 0. This cutoff is related to the single-electron nature of the considered mechanism.
(ii) Purcell effect on the dipole radiation. The power radiated by the dipoles is modified by the presence of the metal electrodes. The strength of this effect is given by the photon energy-dependent density of optical states (LDOS) in the nanogap, normalized to that of vacuum *ρ*_gap_/*ρ*_0_(*E*).
(iii) Radiation to the far-field. The power radiated by the dipoles outcouples directly to the far-field, or couples to the electrodes. In the second case, part of it is lost through non-radiative processes, and the other part is outcoupled to the far-field. The efficiency of the outcoupling process is given by the photon energy-dependent radiative efficiency *η*_rad_(*E*) of the electrodes.

The electroluminescence spectrum is then given by:

(1) $$\eta_{\text{ext}}\left(E\right)=\eta_{\text{IET}}\left(E\right)\frac{\rho_{\text{gap}}}{\rho_{0}}\left(E\right)\eta_{\text{rad}}\left(E\right)$$

From eq. (1), it comes that the electroluminescence spectrum can be tuned by two means. First, in a static way by suitably tailoring the morphology of the electrodes. This enables controlling the plasmonic response of the nanogap structure and thus the energy dependence of *ρ*_gap_/*ρ*_0_ and *η*_rad_. Second, in a dynamic way by adjusting *V*_bias_. Since *η*_IET_ = 0 if *E* > e*V*_bias_, photons are emitted only at energies smaller than e*V*_bias_. Therefore, increasing *V*_bias_ enables extending the bandwidth of electroluminescence toward higher photon energies.

The corresponding external quantum yield is obtained by integrating *η*_ext_(*E*) over the whole range of photon energy. Achieving a high external quantum yield requires tuning the morphology of the electrodes so that they enable a suitable trade-off between (1) a large fraction of electrons tunneling inelastically, (2) a strong Purcell effect in the nanogap, and (3) an efficient antenna behavior ensuring an optimal outcoupling of the generated optical power to the far-field.

For the case of a scanning tunneling microscope traveling over a metal surface, the physical mechanism governing light emission can be understood as follows. When the tip-sample separation distance is around 0.5–1 nm, the surface plasmons on the two surfaces strongly interact and form a coupled plasmon mode. Light emission is resonantly enhanced due to the formation of the tip-induced plasmon mode in the cavity formed between the tip and sample. The resonance in the emission spectrum occurs at the frequency for which half a wavelength of the coupled mode fits into this cavity. The charge oscillations associated with the coupled plasmon mode have opposite signs on the two electrodes. This has two major consequences: (i) the electromagnetic fluctuations are enhanced since the charge on the first surface give a field that polarizes the second surface, so that the polarizing field at the first surface increases. (ii) The coupled plasmon mode is locally charge neutral which red-shifts the resonance peak relative to the surface plasma frequency. It is worth noticing that the effects of retardation are small for sample materials such as Au or copper and tip materials such as tungsten or iridium [63]. Recently, a STM has been employed to locally modify the surface of a Si/Au film stack via heating, which was enabled by a high-density tunnel current. Using this technique, hybrid Si/Au nanoantennas with a minimum diameter of 60 nm have been formed, resulting in structures that efficiently emit photons in the visible range owing to the inelastic tunneling of electrons through the junction [64].

Considering the theory of photon emission in electron tunneling to metal nanoparticles, one can distinguish between inelastic tunneling where the excitation occurs when the electron is in the vacuum barrier and the hot-electron mechanism, where a tunneling electron injected in the particle excites the surface plasmon. For a particle with a few ten nanometer radius, the hot-electron mechanism is quite ineffective and light emission is governed by inelastic tunneling. But for very small particles, *R* < 1.5 nm, light emission is dominated by the hot-electron injection mechanism [65].

The properties of surface plasmons excited by electron tunneling near metallic structures have been also examined. A complete calculation is given for surface plasmons localized by particles with curvature near the junction, including the radiation spectrum of the system [66].

### 4.2 Overview of pioneer and recent works

#### 4.2.1 Pioneer works: metal-insulator-metal junctions

Electroluminescence due to inelastic electron tunneling in a nanogap was first observed in the 70s by Lambe and McCarthy in layered metal–insulator–metal nanogap structures [62]. In these structures, the electrodes consisted of noble metals (Au, silver, or aluminum). These early studies showed the potential of such structures as versatile electrically driven light sources emitting in the infrared and visible regions. It was shown that the spectral features of their electroluminescence can be tuned by:

(i) Varying the applied voltage, as shown in Figure 4(b). Increasing the voltage broadens the emission spectrum from the near infrared to the visible, due to the change in *η*_IET_(*E*).
(ii) Controlling the radiation efficiency of the structure. This was achieved by several means, such as tailoring the metal roughness [62, 67, 68], adjunction of a prism [69] or a grating [70]. This also enables tuning the emission pattern of the emitted light.

However, most of these structures presented a relatively large footprint and low values of *η*_ext_ ∼ 10^−6^, far from the theoretical limit estimated to be $∼10^{-1}$ [62].

#### 4.2.2 Recent works: nanogap antennas

The recent progresses of nanofabrication techniques have fostered the design of smaller footprint nanogap structures showing electroluminescence due to inelastic electron tunneling. They combine a strong Purcell enhancement with a more efficient outcoupling of the generated optical power. These structures, which are called ’nanogap antennas’, besides presenting a higher external electroluminescence quantum yield than standard layered metal–insulator–metal nanogap structures, also present an excellent potential for controlling the spectrum, pattern and polarization of the emitted light.

Hereafter, the recently reported nanogap antennas are grouped into two classes: in-plane nanogap antennas, in which the metal electrodes are in the same plane and vertical nanogap antennas, which rely on vertical integration. In these two classes, the electrodes consist of noble metals. Finally, nanogap antennas including electrodes based on alternative materials, i.e. others than noble metals, are presented. The advantages and drawbacks of each type of nanogap antennas are discussed, taking into account several aspects: external quantum yield, radiated power, spectral, spatial and polarization properties of the electroluminescence, footprint, integration, and scalability. The related applications are also discussed. The key features of selected nanogap antennas are gathered in Tables 1 and 2.

**Table 1:** Main features of vertical nanogap antennas selected in this review: key geometrical features, main materials, main fabrication methods, nanogap features (gap width, barrier height, conductance), external electroluminescence quantum yield *η*_ext_ main results. In all these works, light emission was governed by inelastic electron tunneling together with plasmon assisted radiation. Unless otherwise stated, measurements were done at room conditions.

Antenna	Materials	Fabrication	Gap width barrier	*η*_ext_	Main results	Ref.
geometry			conductance			
NH array	Au NH/BN/Au	FIB, BN transfer	3 nm – –	2.10^−5^	Spectral tuning VIS-NIR Polarization control 200 MHz modulation	[79]
Tunnel junctions	Gr/h-BN/Gr	UV lithography, BN, transfer	2–4 nm – –	6.10^−7^	Twist-angle-controlled emission	[80]
	Al/AlO_*x*_/Cu	EBL	2–3 nm – –	10^−5^–10^−7^	Spectral tuning VIS-NIR Long operation lifetime 18 h	[84]
Crossed capped NR-NST	Ag NW/cap/Au NST	EBL Drop-cast	1.7 nm 2.5 eV –	2.10^−5^	Spectral tuning NIR (200 nm) Narrow spectrum (50 nm)	[81]
NW on metallic QW	TiN/Al_2_O_3_ Ag NRs	Sputtering, seed-mediated synthesis	– 8 eV –	0.3	The highest *η*_ext_ Radiated power 2 nW	[82]
Capped NP on two NSTs	Au NP/cap/Au NST	EBL Templated ET	1.8 nm – –	2.10^−4^	Spectral tuning NIR Flexible approach Larger scale compatible	[83]
NS on thin stack	Au NS/Gr/Al_2_O_3_/Au	EBL CVD ALD	2–4 nm – –	10^−6^	Spectral tuning VIS-NIR Polarization control Easy electrical connection Accurate gap control	[87]
	Ag NS/Gr/BN/Au	Exfoliation Drop-cast	2.3 nm – –	–	Narrow spectrum NIR (50 nm) Decoupling electronic and optical effects	[88]
Capped NP on thin stack	Au NP/cap/Au/Al_2_O_3_/Si	EBL ALD Drop-cast	2–10 nm – –	–	Electroluminescent detection of molecules in the NIR	[89]
Top electrode on vertical NRs	EGaIn/PLH/Au NRs	Templated growth in anodic Al_2_O3	1.1 nm – –	10^−6^	Electrochemical and photochemical tuning	[92, 93]

NP, nanoparticle; NF, nanoflake; NR, nanorod; NW, nanowire; NS, nanostructure; Gr, graphene; BN, boron nitride; NST, nanostripe; EGaIn, eutectic gallium indium; FIB, focused ion beam; AFM, atomic force microscopy; EBL, electron beam lithography; EM, electromigration; ET, electrophoretic deposition; ALD, atomic layer deposition.

**Table 2:** Main features of in-plane nanogap antennas selected in this review: key geometrical features, main materials, main fabrication methods, nanogap features (gap width, barrier height, and conductance), external electroluminescence quantum yield *η*_ext_ main results, and light emission mechanism. Unless otherwise stated, measurements were done at room conditions.

Antenna	Materials	Fabrication	Gap width,	*η*_ext_	Main results	Ref.
geometry			barrier and			
			conductance			
**Light emission mechanism: inelastic electron tunneling, plasmon assisted radiation**						
Capped NP between two NFs	Au NF-cap-Au NP-cap-air-Au NF	FIB AFM	1.3 nm 2.6 eV –	3.10^−4^	Spectral tuning NIR	[71]
Two NRs (V-shaped)	Au NR-air-Au NR	EBL EM	1.1 nm 2 eV –	–	Spectral tuning NIR Directional emission tuning	[72]
Capped NP in bowtie NW	Au NW-cap-Au NP-cap-Au NW	EBL ET	– – <10^−3^ G_0_	–	Sharp Fano resonance NIR^a^	[74]
Bowtie NW with nano-constriction	Au NW-air-Au NW	EBL EM	0.6 nm 3.6 eV 0.2 G_0_	1.10^−4^	Spectral tuning VIS-NIR Radiated power ∼1 nW Operation lifetime 10 h	[75]
2 capped parallele-pipedic NSs	Ag NS-cap-Au NS	Polymer mediated assembly	1.5 nm – –	2.10^−2^	Spectral tuning VIS-NIR High *η*ext Radiated power ∼10 pW	[76]
**Light emission mechanism: elastic tunneling forms hot electron gas (2000 K), blackbody radiation**						
NW with nano-constriction	Au-air-Au	Anodic Al_2_O_3_ EBL, EM	– – 0.8 G_0_	10^−11b^	Synapse-like response in the NIR Overbias emission tail VIS Directional emission tuning	[100]
			–	10^−7b^	Coupling plasmonic waveguide	[101]
Pads with nano-constriction			– – 0.9 G_0_	–	Coupling dielectric waveguide	[103]
**Light emission mechanism: inelastic tunneling generates plasmons, plasmons generate hot**						
**carriers (2000 K), plasmon-assisted hot carrier radiative recombination**						
Bowtie NW with nano-constriction	Au-void-Au AuPd-void-AuPd Pd-void-Pd	EBL EM	Many devices up to 0.25 G_0_	–	Full spectrum overbias VIS for Au-void-Au if no Cr wetting layer^a^	[104]
**Light emission mechanism: single- and multi-electron processes**						
Bowtie tunnel junction	Al-air-Al	EBL	0.1–1 G_0_	–	Tuning light emission through different decay mechanisms	[106]

^a^Measured in vacuum at ∼5 K. ^b^Underestimated due to limited measurement bandwidth. NP, nanoparticle; NF, nanoflake; NR, nanorod; NW, nanowire; NS, nanostructure; FIB, focused ion beam; AFM, atomic force microscopy; EBL, electron beam lithography; EM, electromigration; ET, electrophoretic deposition.

### 4.3 In-plane nanogap antennas

#### 4.3.1 Nanoparticle in a gap and nanorod dimers: tuning the electroluminescence spectrum and pattern

**Figure 5:**
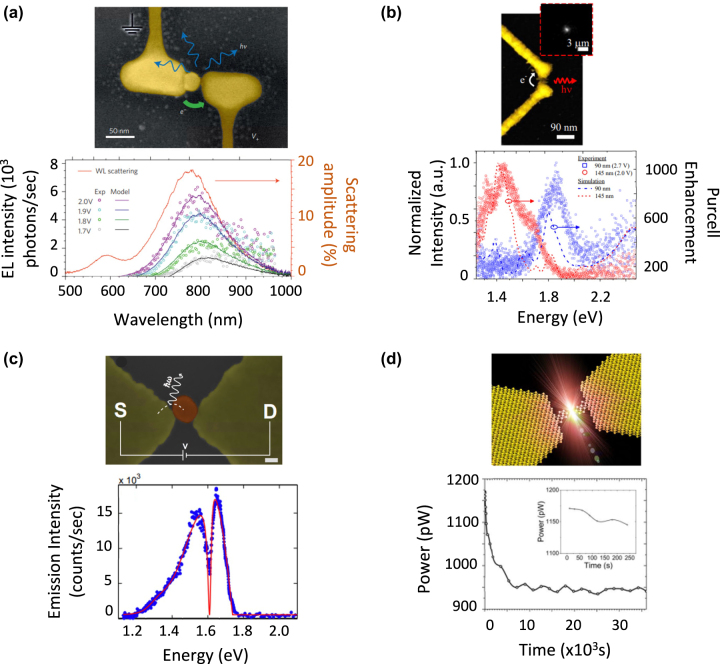
Examples of in-plane nanogap antennas showing inelastic electron tunneling-induced electroluminescence: tuning the emission spectrum, pattern and optimizing the radiated power. (a) An electrically connected single-crystalline gold nanoantenna loaded with a coated gold nanoparticle on a glass substrate. An electroluminescence signal was observed upon applying a voltage. Reproduced from ref. [71] with permission; copyright 2015 Nature Publishing. (b) V-shaped antenna consisting of two Au nanorods. Light is emitted in a preferential direction set by the antenna’s asymmetry. The emission spectrum is dominated by a plasmonic feature that can be tuned in the VIS-NIR range by changing the nanorod length. Reproduced from ref. [72] with permission; copyright 2017 American Chemical Society. (c) Capped Au nanoparticle in a Au bowtie nanowire antenna. A sharp Fano resonance is seen in the NIR due to interference between the narrow plasmon resonance of the nanoparticle and the broad plasmon resonance of the bowtie. Reproduced from ref. [74] with permission; copyright 2016 American Chemical Society. (d) Bowtie Au nanowire antenna with nanoconstriction. This configuration enables the radiated optical power to reach 1 nW, with an operation lifetime of 10 h. Reproduced from ref. [75] with permission; copyright 2019 American Chemical Society.

The first demonstration of spectrally tunable electroluminescence by electrically driven in-plane nanogap antennas was reported in 2015 by Kern et al. [71] Each antenna consisted of two single-crystal Au nanoflakes with the same orientation separated by a 25 nm gap, which were connected to the external voltage supplied by metal nanowires as shown in Figure 5(a). These structures were fabricated by focused ion beam lithography. A Au nanoparticle capped by a ligand shell was then brought between the two nanoflakes with an atomic force microscopy tip, until it touched one of the nanoflakes and was separated from the other by a nanogap. Upon applying a voltage, an electroluminescence signal was observed. The corresponding spectrum showed a resonant structure in the near infrared attributed to the LSP of the nanoflakes. Upon increasing the voltage up to 2 V, this band was shown to shift toward higher photon energies. In addition to this dynamic tuning, the electroluminescence spectrum was tuned by controlling the location of the Au nanoparticle within the gap. The electroluminescence of the fabricated structures showed a symmetric dipole-like pattern.

Later, Gurunarayanan et al. fabricated in-plane nanogap antennas based on polycrystalline Au nanorods [72]. Electron beam lithography was used to fabricate a nanorod dimer connected by a nanoconstriction, which was then submitted to an electromigration process to form a nanogap between them. In contrast with the approach by Kern et al., it was not needed to bring any nanoparticle to form the nanogap. Moreover, the nanorods were tilted one with respect to the other to form a “V-shaped antenna”, as shown in Figure 5(b). The corresponding electroluminescence spectrum was shown to present a LSP-related resonance which was tuned from the near infrared to the visible by varying the nanorod length. Furthermore, an asymmetric electroluminescence pattern, with most of the power being radiated in a tilted direction, was achieved. Such directional emission was attributed to the far-field interference of the dipolar emission from the gap and the quadrupolar-like LSP scattering from the antenna. The strength of this interference was shown to depend on the antenna structure (length and orientation of the nanorods) and on the applied bias, thus allowing the tuning of the electroluminescence pattern both by design and dynamically.

In a more recent work, the directionality of light emission has been numerically addressed in an electrically driven nano-strip MIM tunnel junctions. The device consisted of an Ag–SiO_2_–Ag stack with SiO_2_ of thickness 3 nm, the top electrode of the stack was divided into 16 nano-strips, with two of the tunnel junctions at the center acting as source. The emission was tuned to desired angles by appropriate selection of the periodicity and the excitation source. The mechanism governing this directional emission was the constructive interference of directly emitted photons from the primary and secondary sources, with negligible contribution from the scattered surface plasmons [73].

It is worth noting that, in the three previous works, the electroluminescence spectra presented relatively broad features, with a linewidth greater than 100 nm. Achieving sharp features may be useful for applications such as pure color emission or sensing. In this context, Vardi et al. showed that sharp Fano resonances can be produced in the electroluminescence spectrum of in-plane nanogap antennas [74]. They achieved such a resonance in the near infrared by introducing a colloidal Au nanoparticle capped by an organic layer in the gap of a bowtie nanowire, as shown in Figure 5(c). The bowtie nanowire was fabricated by electron beam lithography and the nanoparticle was introduced by electrostatic trapping. It was shown that the Fano resonance results from the interaction between the narrow LSP resonance of the nanoparticle and the broader LSP resonance of the bowtie.

#### 4.3.2 Parallelepipedal nanostructures dimer: maximizing the external electroluminescence quantum yield

The external electroluminescence quantum yield of the antennas reported in the previous works reached at most values of *η*_ext_ ∼ 10^−4^ under few-volt applied bias. This is much higher than with the early layered metal-insulator-metal nanogap structures, but still far from the theoretical limit. In this context, Qian et al. reported a nanogap antenna design enabling *η*_ext_ to reach much higher values [76]. Their design consists of a dimer of single-crystal Ag nanostructures with a parallelepipedal shape. These nanostructures are encapsulated by an ultrathin polymer layer. A polymer-mediated assembly process is followed to achieve an edge-to-edge orientation of the nanostructures with an ultrathin nanogap. The electroluminescence spectrum of the antenna showed LSP-related resonant features tunable in the visible and near-infrared by controlling the nanostructure shape. By accurately adjusting their three axis lengths, the authors achieved a resonant response for *η*_rad_ and *ρ*_gap_/*ρ*_0_ at the same photon energy. Combining these features altogether enabled *η*_ext_ to reach values in the range of 10^−2^ under a ∼3 V bias.

#### 4.3.3 Bowtie with nanoconstriction: maximizing the electroluminescence power and operation lifetime

The quantities that are truly relevant for benchmarking the performance of a light source are the optical power it radiates and its operation lifetime. For the nanogap antennas with the highest reported *η*_ext_ ∼ 10^−2^, the radiated optical power was only in the range of 10 pW [76]. This points a weak current of inelastically tunneling electrons, with values typically in the range of tens to hundreds of nA under a few-volt bias for these structures. In addition, the operation lifetime of the antennas was not studied.

Recently, Qin et al. fabricated a nanogap antenna structure aiming at combining a relatively high *η*_ext_ with a strong tunnel current and studied its operation lifetime [75]. The nanogap antenna was produced by shaping a bowtie in a single-crystal Au nanowire by electron beam lithography. An ultrasmall nanogap, shown in Figure 5(d), was formed at the bowtie’s center by a controlled electromigration process. The ultrasmall nanogap volume enabled a tunnel current in the range of tens of μA under a few-volt bias. In addition, because of the bowtie’s LSPs, the antenna’s *η*_rad_ and *ρ*_gap_/*ρ*_0_ were relatively high so that its *η*_ext_ reached 10^−4^. Consequently, the radiated optical power in the VIS-NIR reached values near 1 nW. The power remained at this value during 10 h, pointing at the excellent operation lifetime of the antenna.

More recently, an optical-field-controlled emission of electrons in the ultrafast regime at the metal–vacuum interface was demonstrated [77, 78]. When a strong driving pulse illuminates the nanoantenna junction, a large local electric field is generated at the nanoantenna tip. Due to the combined effect of the geometric field enhancement resulting from the sharp radius of curvature and the localized surface plasmon polariton in the antenna, the locally enhanced field can exceed the incident electric field of the driver pulse by more than one order of magnitude depending on the spectral overlap with the plasmonic resonance. If high enough, the local electric field substantially bends the surface potential, and a net tunneling current of electrons flows through the potential barrier resulting in an optical-field-controlled emission of electrons at the metal–vacuum interface. Owing to the strong nonlinearity of the emission process, the electron bursts generated in the device were on the order of several hundred attoseconds for the case of NIR fields.

### 4.4 Vertical nanogap antennas

#### 4.4.1 Nanohole array: tuning the electroluminescence spectrum and polarization

**Figure 6:**
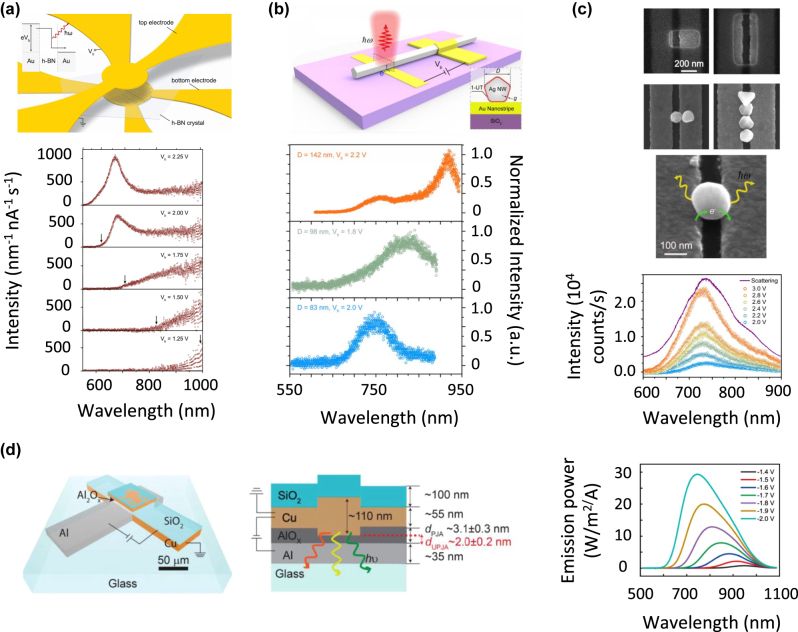
Example of vertical nanogap antennas showing inelastic electron tunneling-induced electroluminescence. (a) Hexagonal boron nitride tunnel junctions. Upon applying a bias voltage, inelastic electron tunneling occurs and the optical power of the generated nanogap dipoles is radiated to the far-field by the nanohole LSPs. Reprinted with permission from ref. [79] with permission; copyright 2015 Nature Publishing. (b) Crossed nanorod-nanostripe antenna. A capped silver nanorod is deposited on top of an Au nanowire. The electroluminescence spectrum shows narrow resonances arising from gap plasmons between the nanorod and nanowire. The resonance is tuned in the NIR by varying the nanorod diameter. Reproduced from ref. [81] with permission; copyright 2019 American Chemical Society. (c) Capped Au nanoparticle trapped on two Au stripes fabricated by templated electrophoretic trapping. This is an up-scalable approach that enables accurately locating one or several nanoparticles over the stripes to enable a tunable plasmon-enhanced electroluminescence. Reproduced from ref. [83] with permission; copyright 2019 American Chemical Society. (d) Schematic illustration of an Al–AlO_*x*_–Cu tunnel junction with a cross-section profile of the junction for a longer operation lifetime. Spectral photon emission power collected at various voltages. Reproduced from ref. [84] with permission; copyright 2022 Wiley-VCH GmbH.

These advantages were first demonstrated by Parzefall et al., who produced vertical nanogap antennas in which the electroluminescence is outcoupled with nanohole Au structures [79]. In this design, an Au thin film acting as first electrode is first grown. This film is structured by focused ion beam lithography to design a nanohole array. A boron nitride (h-BN) ultrathin film is then deposited onto this structured film. Finally, a continuous Au thin film is grown onto the boron nitride film, to act as second electrode (Figure 6(a)). In this embodiment, the nanogap is thus the space between the structured and continuous Au films and its thickness is controlled by tuning the boron nitride thickness. Upon applying a few-volt bias between the electrodes, inelastic electron tunneling occurs and the optical power of the generated nanogap dipoles is radiated to the far-field by the nanohole LSPs. Therefore, the electroluminescence spectrum can be tuned from the near-infrared to the visible by changing the nanohole dimensions, in addition to varying the voltage. For structures with rectangular nanoholes, the emitted light was linearly polarized. *η*_ext_ values up to 2 × 10^−5^ were achieved.

In a more recent work, the same group has demonstrated twist-controlled resonant light emission from graphene/hexagonal h-BN/graphene tunnel junctions. The tunnel junctions consisted of two twist-angle-controlled single-layer graphene electrodes separated by a h-BN tunnel barrier of 7–12 atomic layers [80]. The device was supported by a transparent glass substrate, and the graphene layers were connected to gold electrodes. For small twist angles, the emission spectrum featured a pronounced resonance, which was also tunable with the applied bias. In nearly aligned devices, approximately 40 % of the photons was emitted on resonance. *η*_ext_ values of only 6 × 10^−7^ were recorded, but can be enhanced further by optical antennas.

#### 4.4.2 Crossed nanorod-nanostripe antennas: tunable electroluminescence spectrum with narrowband features

To achieve narrowband electroluminescence, He et al. fabricated vertical nanogap antennas by stacking a single-crystal colloidal silver nanowire on an Au nanostripe with a perpendicular orientation, as shown in Figure 6(b) [81]. The width of the nanogap is determined by the thickness of a molecular layer capping the silver nanowire. In the nanogap, the inelastically tunneling electrons excite gap LSPs, and the power is then radiated to the far-field. The electroluminescence spectrum is thus dominated by resonant features related to the gap LSP modes, which can present a narrow linewidth and can be spectrally tuned by changing the nanowire diameter. Electroluminescence spectra with linewidth of few tens of nm and a 200-nm tunability range in the near infrared were achieved.

In a more recent work, Qian et al. has found a way to enhance the low surface plasmon excitation efficiency by introducing resonant inelastic electron tunneling at the visible/near-infrared frequencies, therefore demonstrating highly-efficient electrically-driven surface plasmon sources [82]. The low efficiency of surface plasmon excitation is due to the fact that elastic tunneling of electrons is much more efficient than inelastic tunneling. In this work, resonant inelastic electron tunneling has been supported by a TiN/Al_2_O_3_ metallic quantum well heterostructure, while monocrystalline silver nanorods were used for the surface plasmon generation. The radiated power was clearly boosted owing to the large tunneling current with values up to 2 nW. An EQE up to 30 % was also observed and was claimed to be the highest value demonstrated thus far for an on-chip electrically-driven surface plasmon generation.

#### 4.4.3 Nanoparticle trapping on metal stripes: toward higher throughput fabrication

The two previous antenna designs are not ideal in view of a high-throughput fabrication. Focused ion beam lithography was used to produce the nanohole arrays and positioning reproducibly a nanowire on a nanostripe with a perpendicular orientation is a challenging task. In this context, He et al. demonstrated a scalable method to accurately locate Au nanoparticles forming nanogaps with the top surfaces of two parallel Au nanostripe electrodes [83]. A PMMA layer was first deposited onto the electrodes, and a cavity was engineered in this layer by lithography. The cavity was designed to overlap with the two electrodes, as shown in Figure 6(c). Finally, a solution of nanoparticles was casted onto the structure and an AC voltage was applied between the electrodes. This resulted in trapping nanoparticles within the cavity. With a suitable cavity design, a nanoparticle capped by an organic layer can form nanogaps with the two electrodes. The thickness of the capping layer sets the nanogap width. Antennas fabricated following this method displayed *η*_ext_ values up to 2 × 10^−4^ with tunneling currents in the range of few tens of nA. Moreover, by designing adequate cavities, nanoparticle dimers or chains can be located above the electrodes. This enables a broad tuning of the electroluminescence spectrum in the near infrared region.

#### 4.4.4 AC-driven Al–AlO_*x*_–Cu tunnel junction: toward longer operational lifetime

Most plasmonic tunnel junctions are studied under DC operation with a relatively large bias (and associated currents) to observe light emission at optical frequencies. Large voltages risk device failure and reduce device lifetimes. This challenge was tackled by Wang et al. [84] in their recent paper by using MIM junctions based on Al and Cu materials. The AlO_*x*_ layer defined the tunnel barrier thickness, the Al electrode functioned as the bottom electrode, and the Cu electrode formed the top electrode. Light emission mechanism was attributed to inelastic electron tunneling, where the electrons tunnel through the AlO_*x*_ barrier and lose energy to all available optical modes. Electron-photon conversion efficiencies *η*_ext_ were on the order of 10^−5^–10^−7^. More importantly, the operational lifetime of AC-driven plasmonic tunnel junctions was improved by a factor of three compared to their DC-driven counterparts. Under DC conditions, slow processes that may lead to device failure can readily occur, in particular undesirable electromigration leading to shorts, thus limiting the device operation time to 9.2 h. However, under AC operation, such processes are slow with respect to the voltage changes prolonging the operation time beyond 18.0 h.

Recently, the same group has reported a new structure based on plasmonic tunnel junctions and Schottky diodes that improved the device efficiency [85].

### 4.5 Advantages and drawbacks of in-plane nanogap antennas and vertical nanogap antennas

In sum, both in-plane nanogap antennas and vertical nanogap antennas present several advantages that have been described in the previous sections:

– They can operate as electrically driven light sources, emitting in the visible and/or near infrared.
– Their emission spectrum and pattern can be tuned by properly designing the antenna morphology, and dynamically by adjusting *V*_bias_.
– Broad and very narrow spectral features can be achieved.
– They can efficiently operate under a relatively low bias *V*_bias_ of few volts. Under such bias, external electroluminescence quantum yields up to *η*_ext_ ∼ 10^−2^ and radiated optical powers in the nW range were produced.
– Operation lifetimes up to 10 h were reported.

Another important advantage intrinsic to in-plane nanogap antennas is that each antenna can be connected individually to a voltage supply. This is appealing for the design of multiplexed nanoscale light sources.

Beyond these peculiarities, in-plane nanogap still needs to be optimized to produce reliable nanostructures with a high production throughput to be exploited in real-world devices. The current fabrication methods of in-plane nanogap antennas suffer in most of the cases from [86]:

– A low production throughput, which is especially notorious for electron beam and focused ion beam lithography, atomic force microscopy positioning, and electromigration. It is worth noting that, in many cases, lithography with a nanoscale precision is needed to fabricate the electric contacts connecting the antenna to the voltage supply.
– A limited reproducibility, especially in the control of the nanogap width, which will impact the repeatability of the tunnel current, *η*_ext_ and the radiated optical power. This is especially applicable for the approaches that rely on electron beam lithography and/or electromigration for the nanogap design. These methods introduce a surface roughness that is difficult to reproduce exactly from one experiment to another, thus leading to an uncertainty in the nanogap width.

Vertical nanogap antennas enable relieving some of the fabrication drawbacks of in-plane nanogap antennas. In particular, the electrical contact with the voltage supply can be fabricated with no need of high-accuracy lithography tools. Thus, vertical nanogap structures can have a higher production throughout percentage compared to their in-plane nanogap counterparts and tackle the main challenge of scalability. Moreover, nanogaps of vertical nanoantennas consist in most cases of a spacer layer whose thickness can be accurately controlled and reproduced.

### 4.6 Nanogap antennas with alternative materials

Nanogap antennas with electrodes consisting of other materials than noble metals (graphene, silicon, eutectic gallium-indium) were also shown to exhibit inelastic electron tunneling–induced electroluminescence. Using these alternative materials presented advantages such as the decoupling between electrical and optical properties (graphene), a practical electrical connection (graphene), electroluminescence and sensing on a silicon platform (silicon), or a reversibly tunable electroluminescence driven by photochemical or electrochemical processes.

#### 4.6.1 Graphene-based antennas: decoupling electrical and optical properties, practical electrical connection

**Figure 7:**
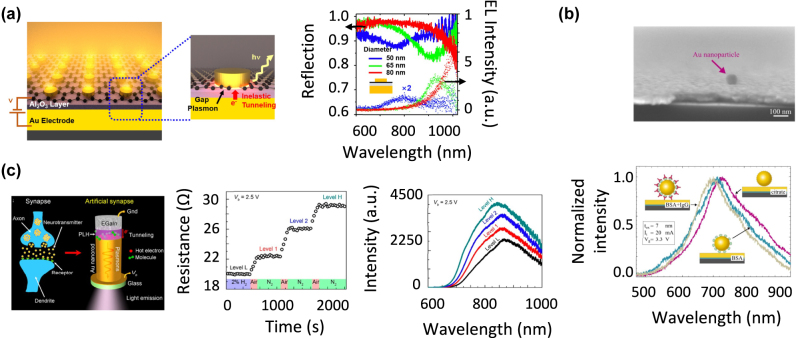
Example of vertical nanogap antennas based on alternative materials and showing inelastic electron tunneling-induced electroluminescence. (a) Graphene-based antennas. Inelastic tunneling occurs between the Au bottom electrode and the top graphene electrode. Au nanostructures over the graphene sheet enhance the LDOS in the gap thanks to gap plasmons, together with the outcoupling of the emitted radiation. The electroluminescence spectrum shows resonant plasmonic features that can be tuned in the NIR by varying nanostructure dimensions. Reproduced from ref. [87] with permission; copyright 2018 American Chemical Society. (b) Silicon-based antennas. Tunneling occurs between a Si film and an Au film separated by a thin aluminum oxide layer. The electroluminescent power is outcoupled by capped Au nanoparticles at the surface of the Au film. The electroluminescence spectrum shows a NIR plasmonic resonance band that is sensitive to the nature of the molecules capping the nanoparticles. This is useful for molecular detection. Reproduced from ref. [89] with permission; copyright 2016 American Chemical Society. (c) Eutectic-gallium–indium based antennas. Tunneling occurs between Au nanorods and a eutectic gallium indium electrode, separated by a redox polymer. Partial and reversible oxidation and reduction of this polymer is achieved electrochemically or photochemically and enables the tuning of the NIR electroluminescence intensity in a synapse-like way. Reproduced from ref. [90] with permission; copyright 2020 American Chemical Society.

Graphene is an electrical conductor with an excellent optical transparency in the visible and near infrared. Therefore, it was used as a transparent electrode in vertical nanogap antennas consisting of a stack including an Au thin film as bottom electrode, a thin aluminum oxide [87] or boron nitride insulating layer [88] and a single-layer graphene sheet as top electrode. Finally, Au or silver nanostructures were deposited onto the graphene sheet to form an antenna structure as in the example depicted in Figure 7(a). The major contrast between graphene-based structures and the other metal–insulator–metal structures is that the former allows to partially decouple the electrical and optical properties of the antenna. While the graphene/insulating layer/Au stack drives the electron tunneling properties, the nanogap LDOS *ρ*_gap_/*ρ*_0_ and the radiative efficiency of the antenna *η*_rad_ can be tuned by adjusting the morphology of the Au or silver nanostructures. Electroluminescence spectra with LSP-related resonances were achieved with Au nanorods, and these resonances were tuned in the visible and near infrared by adjusting the nanorod aspect ratio. This also enabled controlling the polarization of the emitted light [87]. Narrow band electroluminescence (linewidth ∼50 nm) was achieved in the near infrared with silver nanocubes [88]. Decoupling the optical and electrical properties thus brings an additional degree of freedom for optimizing the antenna’s performance compared with standard metal–insulator–metal structures. Furthermore, using a graphene sheet as top electrode enables a more practical electrical connection of the antennas to the voltage supply because its implementation does not require high accuracy lithography.

#### 4.6.2 Silicon-based antennas: plasmonic sensing with an electroluminescent silicon platform

The reported silicon-based nanogap antennas present a vertical geometry with a silicon electrode at the bottom and a metal electrode at the top. The outcoupling of the radiated optical power and thus the electroluminescence spectrum and external quantum yield can be tuned by structuring the metal electrode [89, 91] in the same way as with metal–insulator–metal antennas. As shown by Dathe et al., planar Au/insulating layer/silicon structures supporting Au nanoparticles capped by a thin organic layer present a resonant electroluminescence spectrum peaking in the near infrared. This is depicted in Figure 7(b). The resonance energy is driven by the LSP of the Au nanoparticle-Au electrode structure and is thus sensitive to the thickness of the organic layer. Therefore, this structure enabled the selective detection of molecules (citrate, BSA, BSA + IgG) capping the nanoparticles by tracking the electroluminescence signal.

#### 4.6.3 Eutectic gallium indium-based antennas: electrochemical and photochemical electroluminescence tuning

By using conducting eutectic gallium indium as electrode in nanogap antenna structures, tunable inelastic electron tunneling–induced electroluminescence properties were achieved.

Static tuning was achieved by filling the nanogap between eutectic gallium indium and Au electrodes with a self-assembled molecular junction. By using a suitable molecular material, an asymmetric energy barrier was set across the nanogap. By such means, a bias-selective electroluminescence behavior – similar to the one of a diode – was achieved [92]. By tuning the orientation of the molecules, SPPs could be launched directionally [93].

Dynamic electrochemical and photochemical tuning of the electroluminescence power was achieved by Wang et al. in antennas with the structure displayed in Figure 7(c). Each antenna consisted of a vertical Au nanorod separated from the eutectic gallium indium electrode by a nanogap filled with a redox polymer (PLH) [90, 94]. The optical power generated by inelastic electron tunneling was outcoupled to the far-field through the near infrared plasmon modes of the nanorods. The intensities of the tunnel current and electroluminescence were tuned by controlling the oxidation state of the polymer. This control was achieved by introducing the antennas in an oxidizing gas (air) or a reducing gas (hydrogen), while applying a bias or shining light onto them to generate hot electrons from elastic tunneling across the nanogap. The electrically (resp. optically) generated hot electrons enabled the electrochemical (resp. photochemical) oxidation or reduction of the polymer. Remarkably, the polymer can be left in a tunable partial oxidation state and then fully recover its fully oxidized or reduced state. This enables a reversible multilevel tuning of the electroluminescence signal, with a memory effect like that of a synapse [90].

## 5 Hot carrier processes

### 5.1 Overbias emission

In contrast with the works reviewed in the previous section, some other studies have reported nanogap antennas featuring photon emission at energies above the bias (*E* > e*V*_bias_). The origin of this “overbias emission” is still under discussion. Several mechanisms accounting for overbias emission have been already proposed, depending on the experimental configuration considered: excitation of a plasmon by multiple inelastic tunneling electrons followed by outcoupling of the plasmon [95], simultaneous electrical and optical excitation of a plasmon followed by outcoupling of the plasmon [96, 97], hot-hole formation and electron-electron interaction at the junction [98, 99], generation of a hot electron gas of elastically tunneling electrons followed by blackbody radiation from this gas [100–103], excitation of plasmons by inelastic tunneling electrons, followed by relaxation of these plasmons into hot electron-hole pairs that recombine radiatively with the assistance of plasmons [104]. While the three former mechanisms were proposed to account for the trends of scanning tunneling microscopy experiments, the two latter were proposed in the case of in-plane nanogap antennas.

### 5.2 Blackbody emission by hot electrons

**Figure 8:**
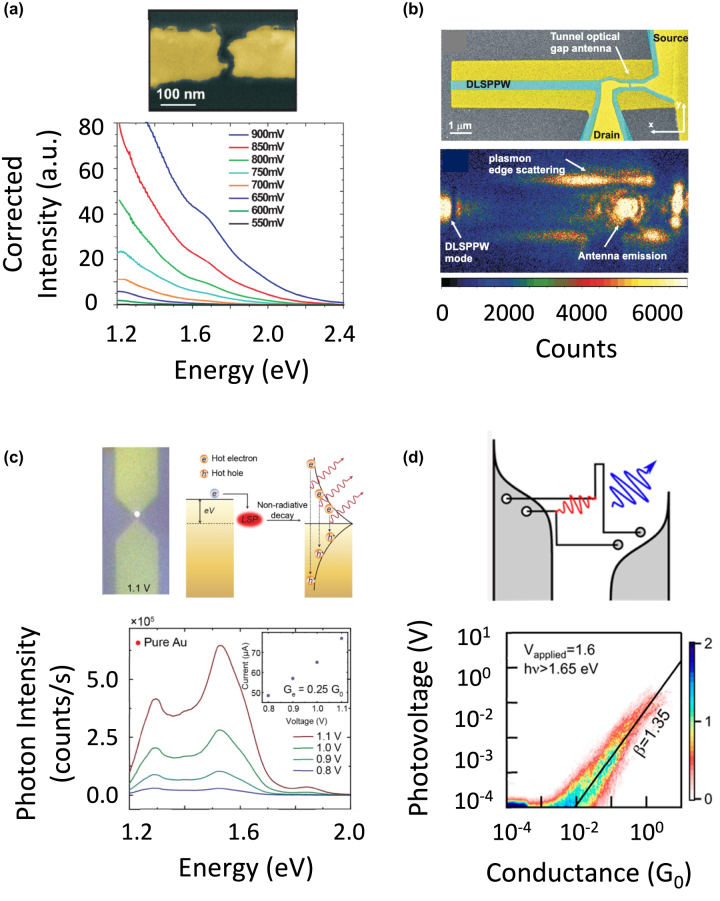
Examples of in-plane nanogap antennas emitting with hot electron – induced light emission. (a) Au nanowire with nanoconstriction. Overbias light emission is observed in the VIS-NIR regime. Reproduced from ref. [100] with permission; copyright 2015 American Chemical Society. (b) SEM image of the dielectric-loaded surface plasmon structure placed on a 2 μm-wide Au strip with the distribution of light in the device when the antenna is polarized with a bias of 1 V and a driving current of 50 μA. Reproduced from ref. [101] with permission; copyright 2016 Optical Society of America. (c) Au bowtie nanowire with nanoconstriction with no chromium wetting layer under the Au film. The whole light emission spectrum appears at photon energies in the NIR together with plasmonic resonance bands. Reproduced from ref. [104] with permission; copyright 2020 American Chemical Society. (d) Illustration of the mechanism for overbias emission from multi-electron processes and 2D photovoltage-conductance histogram of the overbias emission with the mean fitted power law overlaid in black. Reproduced from ref. [105] with permission; copyright 2020 American Chemical Society.

Buret et al. reported overbias emission in Au nanogap antennas produced by direct electromigration of a nanowire fabricated by electron beam lithography. An example of the geometry is shown in Figure 8(a). This allowed the conductance to reach values as high as *G* = 0.8*G*_0_ [100]. For *V*_bias_ in the range of several hundreds of mV, while the electroluminescence peak was in the near infrared, a significant light intensity was measured at overbias energies in the visible. In contrast with antennas for which light emission is driven by inelastic electron tunneling followed by plasmon excitation and outcoupling (previous section), the shape of the emission spectrum was not changed when *V*_bias_ was increased.

Based on a qualitative model, the following mechanism was proposed to account for the experimental trends. Due to the high conductance of the nanogap and its small volume, the density of electrons tunneling elastically through it is remarkably high. Therefore, electron-electron collisions occur at a much faster rate than bulk electron–phonon interactions, which are less likely because of the high surface/volume ratio of the antenna in the nanoconstriction region. This leads to the formation of an electron gas in the metal around the nanogap, with temperatures reaching 2000 K. Its energy is lost radiatively by collisions at the metal surfaces, following a Boltzman curve that extends up to the visible region at energies *E* > e*V*_bias_. Therefore, the overbias emission was attributed in this case to blackbody radiation from a gas of elastically tunneling hot electrons.

Light emission by this mechanism was shown to be compatible with photonic integration. It was coupled to plasmonic waveguides [101] as shown in Figure 8(b), and to dielectric waveguides [103]. Therefore, it is appealing for the design of integrated light sources. Such light sources could present fast modulation capabilities, because the hot electron gas deexcitation processes can occur in times as short as few ps.

### 5.3 Plasmon-assisted radiative recombination of hot electron-hole pairs generated by inelastically-induced plasmons

More recently, Cui et al. studied the overbias emission in nanogap antennas fabricated by electromigration of a nanowire with a bowtie designed by electron beam lithography [104]. By such means the nanogap was located accurately at the center of the bowtie. The typical geometry is shown in Figure 8(c). Many devices were fabricated, showing *G* values up to 0.25*G*_0_. Different materials were used to fabricate the antennas: Au, palladium, or an Au-palladium alloy. In contrast with previous works on electromigrated antennas where a chromium adhesion layer was always introduced under the Au, Cui et al. produced antennas with and without a chromium adhesion layer. Au antennas without chromium showed the strongest overbias emission whereas those with the highest *G* emitted only at photon energies in the visible above e*V*_bias_ and, surprisingly, those with *G* as small as 10^−3^ *G*_0_ still showed some overbias emission. Their light emission spectrum showed features characteristic of blackbody radiation together with antenna LSPs. However, the differences in emission intensity between the antennas made of different materials could not be explained by the related changes in *ρ*_gap_/*ρ*_0_.

To account for these trends, it was proposed that light emission in the Au antennas without chromium wetting layer involves hot carriers and plasmonic effects, the latter coming two times into play. In the proposed mechanism, inelastically tunneling electrons excite plasmons at the nanogap surface. These plasmons then generate electron–hole pairs with a high density. These electron-hole pairs thermalize to generate a gas of hot carriers, which recombine radiatively with the assistance of plasmons.

### 5.4 Single- and multi-electron processes for light emission mechanism

Light emission as a function of conductance in a scanning tunneling microscope operating at room temperature and ambient conditions was examined by Fung et al. [105] A spherical Au single-crystal formed by melting 250-nm diameter Au wire and a cut Au wire were used as the two electrodes. It has been shown that overbias emission was due to a mixture of single- and multi-electron processes rather than being due to blackbody radiation (Figure 8(d)). In particular, the power law of the overbias emission implied an increase of electronic temperature of approximately 200 K which cannot be accounted for using blackbody radiation theory, whereas multi-electron processes can produce blackbody-like phenomena at room temperature.

Recently, Zhu et al. proposed to tune the dominant light emission mechanism through single-electron or higher-order multi-electron inelastic tunneling to recombination from a steady-state population of hot carriers by progressively altering the tunneling conductance of an aluminum junction [106]. It has been shown that the dominant emission mechanism was set by a combination of tunneling rate, hot carrier relaxation timescales, and junction plasmonic properties, which could potentially resolve the long-standing debate regarding the relative roles of these candidate mechanisms.

## 6 Toward miniaturized and tunable nano-optoelectronic devices

Direct electrical excitation of plasmonic modes integrates plasmons and electric nano-circuitry which is highly advantageous as it dramatically reduces the overall footprint of the experiment resulting in novel nano-optoelectronic devices. In this context, inelastic electron tunneling offers an alternative nanoscale light source with switching times at the femtoscale, without the need of additional semiconductor materials as for plasmonic organic and inorganic LEDs. In this context, Ochs et al. demonstrated a device for the selective electrical excitation of two distinct plasmonic modes, which makes it the smallest electrically driven light source with switchable polarization states [107]. Mode selectivity is realized by precisely positioning nanoscale excitation sources within the respective modal field distribution.

A nanoscale organic light-emitting antenna was also recently presented as a color- and directionality-switchable point source. The device consisted of laterally arranged electrically-contacted gold nanoantennas with their gap filled by the organic semiconductor zinc phthalocyanine (ZnPc) [108]. Since ZnPc shows preferred hole conduction in combination with gold, the recombination zone relocates depending on the polarity of the applied voltage and couples selectively to either of the two antennas. Thereby, the emission characteristics of the device also depend on polarity.

The emission of graphene can be extended toward telecom and visible spectral range by stacking two graphene layers and twisting one with respect to the other, which results in altering the band structure of the system and, hence, further tuning the output emission. Kuzmina et al. reported visible light emission from tunnel junctions consisting of a twist between two single-layer graphene flakes separated by a thin layer of heaxagonal boron nitride, and excited via inelastic electron tunneling [80]. A twist between the two flakes of graphene alters the band structure of the system, as demonstrated by a visible photoluminescence produced by strongly bound excitons. The emission spectrum was shown to be strongly dependent on the twist angle *θ*. For *θ* close to 0°, the emission spectrum exhibited a resonant spectral peak in the near-infrared range, which broadens with increasing *θ* and eventually disappears.

An additional class of devices exploiting metallic nanogap antennas and molecular junctions was recently demonstrated. The molecular junction geometry consisted of a monolayer of molecules trapped between a single Au nanoparticle and a flat Au film incorporated within an electrode structure [109]. This robust lithography-free molecular optoelectronic device geometry enabled creating molecular junctions with reproducible morphology and electrical response. The well-defined geometry allowed generating optical-frequency voltages ∼30 mV within the molecular junction from 100 μW incident light, and producing photocurrent by optical rectification (>10 μA/W) from only a few hundred molecules.

## 7 Conclusions

In this review, we presented nanogap antennas with gaps in the nanometer and sub-nanometer scale and displayed the impact of quantum tunneling effects on the plasmon resonances of these structures. We investigated light emission in electrically driven nanogap antennas based on inelastic tunneling effect or hot-electron mechanism and demonstrated a spectrally tunable electroluminescence with the applied bias. Such nanogap structures have been proven as a dominant tool for controlling and manipulating light at the nanoscale level, and enhancing the efficiency of photo-detection, light emission and sensing. We then presented the potential applications of electrically driven nanoantennas and discussed some future challenges and opportunities.
